# The Natural History of Epilepsy in 163 Untreated Patients: Looking for “Oligoepilepsy”

**DOI:** 10.1371/journal.pone.0161722

**Published:** 2016-09-22

**Authors:** Sara Gasparini, Edoardo Ferlazzo, Cinzia Grazia Leonardi, Vittoria Cianci, Laura Mumoli, Chiara Sueri, Angelo Labate, Antonio Gambardella, Umberto Aguglia

**Affiliations:** 1 Department of Medical and Surgical Sciences, Magna Graecia University, Viale Europa, Catanzaro, Italy; 2 Regional Epilepsy Center, Bianchi-Melacrino-Morelli Hospital, Via Melacrino, Reggio Calabria, Italy; McGill University, CANADA

## Abstract

The clinical evolution of untreated epilepsy has been rarely studied in developed countries, and the existence of a distinct syndrome characterized by rarely repeated seizures (oligoepilepsy) is debated. The aim of this study is to assess the natural history of 163 untreated patients with epilepsy in order to evaluate whether oligoepilepsy retains specific features. We retrospectively evaluated 7344 patients with ≥2 unprovoked seizures. Inclusion criteria: sufficient anamnestic/EEG data, disease duration ≥10 years, follow-up ≥3 years. Exclusion criteria: psychogenic seizures, natural history of disease <5 years. The 163 included subjects were divided into 2 groups according to seizure frequency: oligoepilepsy (≤1/year; 47 subjects) and controls (>1/year; 116 subjects). We also evaluated seizure frequency during the natural history. There were no differences between groups regarding duration of natural history, family history of epilepsy/febrile seizures, interictal EEG. Subjects with oligoepilepsy differed from controls in terms of sex (females 38% vs. 58%, p = 0.03) and drug resistance (6% vs 28%; p = 0.003). Juvenile myoclonic epilepsy was more frequent in controls (9.5% vs 0%, p = 0.04). Patients with oligoepilepsy, differently from controls, had stable seizure frequency. Oligoepilepsy represents a favourable evolution of different epileptic syndromes and keeps a stable seizure frequency over time.

## Introduction

The clinical evolution of untreated epilepsy has been rarely studied in developed countries, as the widespread availability of effective treatment with antiepileptic drugs (AEDs) makes studies on natural history of epilepsy difficult to realize. Data on untreated patients come from studies in resource-poor countries [1] or studies on patients with a long delay between onset and diagnosis of epilepsy [2–6]. However, none of these studies analysed seizure frequency at onset in order to establish the existence of a condition characterized by rarely repeated seizures (oligoepilepsy), as defined by the 2001 ILAE proposal of a Diagnostic Scheme for People with Epileptic Seizures and with Epilepsy [7]. Even though patients with rare seizures are frequently encountered in clinical practice, in the last 25 years, only three papers described subjects with “oligoepilepsy”[8–10]; of note, authors did not clarify clinical features of epilepsy. In particular, it is not known whether oligoepilepsy constitute a syndrome per se or it represents a benign evolution of different epileptic syndromes.

The aim of the present study is to evaluate the natural history of patients with epilepsy who were untreated for at least five years from onset, in order to look for the existence of oligoepilepsy, to evaluate its main features and to establish if it is a unique syndrome or a benign evolution of different epileptic syndromes.

## Methods

### Data source and subjects evaluation

We own a database collecting data from epileptic patients consecutively observed during almost 30 years, some of them showing a long natural history. We retrospectively evaluated data from 7344 subjects with at least two unprovoked seizures [11], consecutively observed for the first time between April 1987 and July 2011 in our centre. Patients were recruited from general practitioners, paediatricians, emergency department or they referred on their own. As this study did not include any specific intervention on patients and collected data were anonymous, approval from Ethic Committee was not necessary according to local regulations.

### Inclusion and exclusion criteria

Inclusion criteria were: sufficient anamnestic, clinical, imaging and EEG data to make a diagnosis of epilepsy; follow-up of at least three years in our centre; disease duration of 10 years or more. Transient paroxysmal events considered unrelated to epilepsy, including migraine auras, isolated déjà-vu or unspecific dizziness were excluded. Since prior events were recalled by the patients after a long interval, we decided to consider all other brief, self-limiting events as possible seizures, regardless of the physician’s impression on the type of event. All seizures occurring in a strict temporal relationship with known triggers (e.g. alcohol abuse or withdrawal, prolonged sleep deprivation, etc.) were considered as provoked seizures and did not account for the diagnosis of epilepsy. Exclusion criteria were: coexistence of psychogenic non-epileptic seizures (PNES) or psychosis; treatment with any AED during the first five years of disease. In this study, “oligoepilepsy” is defined as epilepsy with seizure frequency of ≤1 seizures per year during the first 5 years of natural history.

### Included variables

The following variables were considered: sex, age at last follow-up, age at onset of epilepsy, disease duration (interval between first seizure and last follow-up visit), family history of epilepsy or febrile seizures (FS) in first and second degree relatives, duration of natural history of epilepsy (interval between first seizure and beginning of AED treatment), EEG activity (normal, abnormal slowing, paroxysmal activity), epileptic syndrome according to 2010 ILAE classification [12] and drug resistance following ILAE definition [13]. All EEGs were accomplished following 10–20 International System. EEGs were performed at different times during patients’ clinical history. Finally, we analysed variations in frequency of seizures within each group: for each patient, we evaluated the frequency of seizures during the first period (five years) and the ensuing period (until AED treatment start) of natural history. In these two periods, seizure frequency was divided in the following categories: ≤ 1 seizure per year; > 1 seizure per year and ≤ 1 seizure per month; > 1 seizure per month and ≤ 1 seizure per week; > 1 seizure per week and ≤ 1 seizure per day; > 1 seizure per day; unknown.

### Statistic analysis

For continuous variables, mean, standard deviation (SD), median and range were calculated. Categorical variables were expressed in terms of percentages. Differences between groups were assessed with Mann-Whitney test and Fisher’s exact test as appropriate. Wilcoxon range test was used for within-group comparison of ordinal variables.

## Results

### Included and excluded subjects

**Fig 1:**
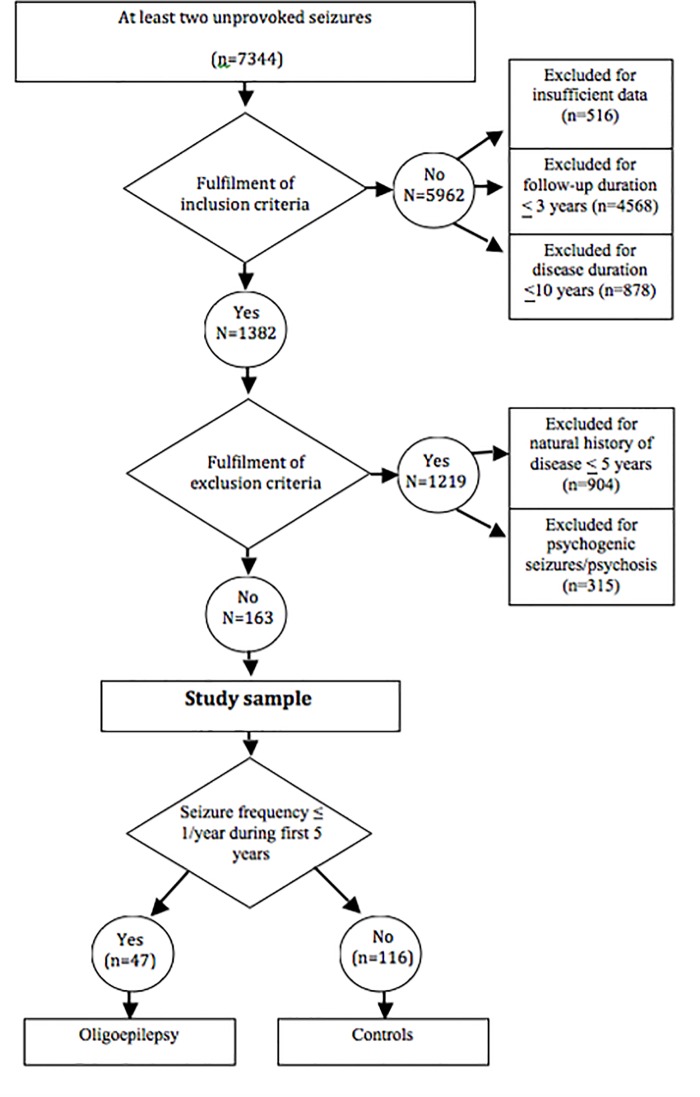
Flowchart of included and excluded subjects.

Of 7344 evaluated patients, 1382 were included in the study. The flowchart of included and excluded subjects is reported in Fig 1. Of these, 1219 were excluded because of AED treatment during the first five years (# 904; 74%) or coexistence of PNES and/or psychosis (# 315; 26%). Hence, study sample included 163 subjects, who were divided in two groups: oligoepilepsy (seizure frequency ≤1 seizure/year during the first five years of natural history of the disease) and controls (>1 seizure/year during the same period).

### Main findings

**Fig 2:**
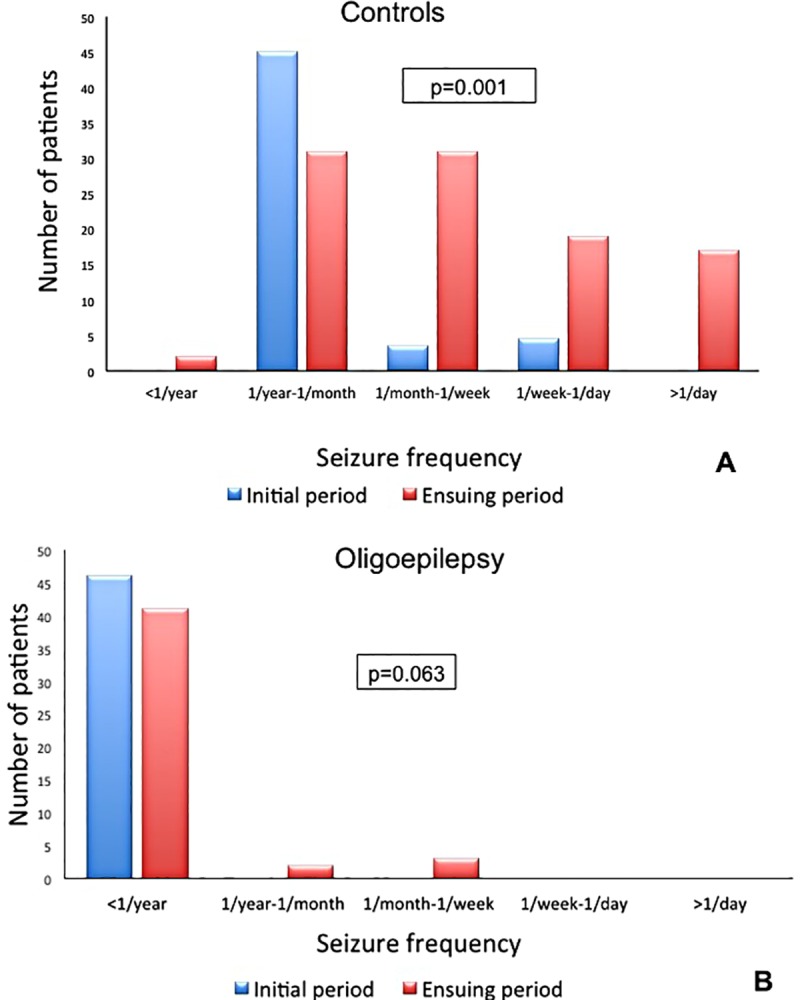
Distribution of seizure frequency during natural history of epilepsy. A: controls, B: oligoepilepsy.

Detailed information about patients with oligoepilepsy (n = 47) and controls (n = 116) are reported in Table 1. Female sex was less represented in subjects with oligoepilepsy compared to controls (38% vs. 58%, p = 0.03). Juvenile myoclonic epilepsy (JME) was significantly less frequent in oligoepilepsy as compared with controls (0% vs. 9.5%, p = 0.04). Patients with oligoepilepsy significantly differed from controls in terms of drug resistance (6% vs. 28%, p = 0.003); reciprocally, a higher proportion of subjects with oligoepilepsy was seizure-free at last control (74% vs. 44%, p<0.001). Of note, the two groups did not significantly differ in terms of distribution of remaining epileptic syndromes and duration of natural history. All subjects with drug-resistant oligoepilepsy were affected by temporal lobe epilepsy associated with hippocampal sclerosis. Seizure frequency during the whole natural history of epilepsy was determined in 46/47 (98%) patients with oligoepilepsy and in 101/116 (87%) controls. Frequency during the natural history varied significantly from initial period and ensuing period (controls: median 6 years, range 1–52; oligoepilepsy: median 5 years, range 1–41 years) in controls (shifting from lower to higher frequency class = 15, shifting from higher to lower frequency class = 2, p = 0.001; Fig 2A) but not in oligoepilepsy group (shifting from higher to lower frequency class = 5, shifting from lower to higher frequency class = 0, p = 0.063; Fig 2B).

**Table 1:** Demographic and clinical data of 47 patients with oligoepilepsy and 116 controls.

	Study sample n = 163	Oligoepilepsy n = 47	Controls n = 116	P value
**Female gender**	85 (52%)	18 (38%)	55 (58%)	**0.03**
**Current age, years: median (range)**	52 (20–94)	50 (23–94)	53 (20–93)	0.91
**Age at onset, years: median (range)**	17 (0–80)	21 (3–80)	17 (0–68)	0.84
**Disease duration, years: median (range)**	25 (10–73)	24 (10–70)	26 (11–73)	0.69
**Family history of epilepsy/FS**	65 (40%)	17 (36%)	48 (41%)	0.60
**Natural history duration, years: median (range)**	11 (5–57)	10 (5–46)	11 (5–57)	0.75
**EEG activity**				
• **normal**	34 (21%)	13 (28%)	21 (18%)	0.20
• **slowing**	45 (28%)	11 (23%)	34 (29%)	0.56
• **paroxysmal activity**	84 (51%)	23 (49%)	61 (53%)	0.73
**Aetiology**				
• **Idiopathic**	26 (16%)	6 (13%)	23 (20%)	0.37
• **Structural-metabolic**	61 (37%)	14 (30%)	47 (41%)	0.59
• **Unknown**	78 (47%)	27 (57%)	46 (39%)	0.06
**Epileptic syndrome**				
** *Determined syndrome*** ^**a**^	160 (97%)	45 (96%)	115 (99.1%)	0.07
• **BCOE**	1 (0.6%)	0	1 (0.9%)	1
• **JAE**	4 (2.4%)	1 (2.1%)	3 (3.4%)	0.32
• **JME**	11 (6.7%)	0	11 (9.5%)	**0.04**
• **Epilepsy with GTCs alone**	2 (1.2%)	1 (2.1%)	1 (0.9%)	0.49
• **Other fTLE**	2 (1.2%)	1 (2.1%)	1 (0.8%)	0.49
• **Reflex epilepsy**	1 (0.6%)	1 (2.0%)	0	0.29
• **Structural/metabolic**	61 (37%)	14 (30%)	47 (41%)	0.22
⋄**Vascular**	9/61 (15%)	4/14 (29%)	5/47 (11%)	0.19
⋄**Neoplastic**	4/61 (6.5%)	0	4/47 (9%)	0.56
⋄**Inflammatory**	4/61 (6.5%)	1/14 (7%)	2/47 (4%)	0.55
⋄**Malformation**	12/61 (19.6%)	1/14 (7%)	11/47 (23%)	0.26
⋄**Degenerative**	1/61 (1.6%)	0	1/47 (2%)	1
⋄**Traumatic**	5/61 (8.2%)	1/14 (7%)	4/47 (9%)	1
⋄**Perinatal insult**	7/61 (11,5%)	1/14 (7%)	6/47 (13%)	1
⋄**Hippocampal sclerosis**	11/61 (18%)	3/14 (21%)	8/47 (17%)	0.70
⋄**Other**	6/61 (6.5%)	3/14 (21%)	5/47 (6%)	0.37
• **Epilepsies of unknown cause**	78 (47.8%)	27 (57%)	46 (43%)	0.06
***Undetermined syndrome***	3 (1.8%)	2 (4%)	1 (0.9%)	0.20
**Drug resistance**				
• **Yes**	36 (22%)	3 (6%)	32 (28%)	**0.003**
• **No**	85 (52%)	35 (74%)	51 (44%)	**<0.001**
• **Undetermined**	42 (26%)	9 (19%)	32 (28%)	0.24

Legend to table.

^**a**^ Only syndromes with at least 1 affected patient are listed. BCOE = benign childhood occipital epilepsy; FS = febrile seizures; fTLE = familial temporal lobe epilepsy; GTCs = generalized tonic-clonic seizures; JAE = juvenile absence epilepsy; JME = juvenile myoclonic epilepsy; SD = standard deviation.

## Discussion

This is the first study that identifies clinical features of “oligoepilepsy”, based on the analysis of a population with a long natural history of epilepsy (15 years on average). Patients with oligoepilepsy represent 0.6% of the population referring to our Centre. Although it is not infrequent to observe patients with rarely repeated epileptic seizures who do not receive AED treatment, in the last 25 years only three papers [8–10] have described the clinical features of patients with “oligoepilepsy”. One study [8] described oligoepilepsies as special evolutions of idiopathic epilepsies in middle-aged and older adults. Seizure frequency at onset or natural history before treatment was not described. Another study [9] reported two children with rare epileptic seizures and defined oligoepilepsy as the occurrence of unprovoked seizures with a frequency of <1/year or 3 over 4–5 years. The small sample does not allow generalization of those observations. Finally, a retrospective study [10] has defined “oligoepilepsy” as the occurrence of ≤2 seizures during the last years of follow-up. The authors identify 47 patients with “oligoepilepsy”, but they considered “major seizures” only and included patients with provoked seizures as well as patients receiving AEDs, thus they did not evaluate the natural history of epilepsy. Of note, none of published studies on the natural history of epilepsy [1–6] evaluated seizure frequency at onset, thus patients with “oligoepilepsy” have not been identified.

Our study has brought three main results. First, oligoepilepsy is not a distinct syndrome, but a favourable evolution that can occur in different epileptic syndromes. Second, in oligoepilepsy the low seizure frequency remains unchanged for a long time. Third, the majority of patients with oligoepilepsy undergo remission with treatment. In this study, subjects with oligoepilepsy were more frequently males. Although some epilepsy syndromes may be influenced by hormonal background [14], the effect of sex and sexual hormones on seizure frequency is not well known and a chance finding cannot be excluded. As regards distribution of epileptic syndromes, JME was not found in oligoepilepsy group. This is in line with the observation that JME characteristically manifests with frequent myoclonic jerks at onset [15]. Occasional studies have addressed the question of the existence of an “oligoepilepsy” syndrome as a distinct entity, with homogeneous clinical features. Some authors [16] apply the term “oligoepilepsy” exclusively to generalized epilepsies, while others [17] do not distinguish between syndromes. Our data suggest that oligoepilepsy may represent a benign evolution of different epileptic syndromes, rather than a unique syndrome. In our study, untreated patients with oligoepilepsy maintained a low seizure frequency during many years, while the same is not true for controls. This result suggests that oligoepilepsy retains its benignity over time. In a paediatric population with “non-syndromic” epilepsy [18], low seizure frequency at onset was not significantly associated with a “benign” outcome. However, the involved population was not comparable with our sample, and the classification of seizure frequency did not distinguish patients with very rare seizures (less than one per year).

In our sample, three patients with hippocampal sclerosis showed an increase in seizure frequency and became drug resistant. Those patients developed “delayed intractability”, as described elsewhere [19]. Patients with oligoepilepsy underwent remission and remained seizure free in a higher proportion compared to controls. This result confirms the favourable evolution of oligoepilepsy, while the role of multiple seizures before treatment onset, and of AED treatment itself, remains unclear. A large prospective study found no differences in terms of seizure freedom between early and delayed treatment of epilepsy [20]. However, some observations suggest that a long interval and/or a large number of seizures before treatment onset are associated with medical intractability [21].

This study has two limitations. First, due to strict inclusion criteria, more than 7000 subjects were excluded. The reduction of included sample may have limited the power of statistical analysis. Second, this study is retrospective, thus the real seizure frequency, especially regarding the less evident ones (e.g.isolated auras), may be underestimated due to recall bias. However, the large availability of efficacious AED treatments would make it unethical to realize a prospective study of natural history of epilepsy.

Even with these limitations, we have described a subset of patients with epilepsy and low seizure frequency during a long natural history; these patients had different epileptic syndromes. We may postulate the existence of adjunctive factors modulating seizure frequency. Future studies may clarify this issue.
